# COVID-19 Vaccine-Induced Acute Perimyocarditis: A Case Report

**DOI:** 10.7759/cureus.31554

**Published:** 2022-11-15

**Authors:** Vishal Phogat, Subash Nepal, Satya Siva Prasad Gadula, Amr Wardeh, Dana Aiello

**Affiliations:** 1 Medicine, Upstate University Hospital, Syracuse, USA; 2 Cardiology, Upstate University Hospital, Syracuse, USA; 3 Radiology, Upstate University Hospital, Syracuse, USA

**Keywords:** mrna-covid-19 vaccine, acute myopericarditis, perimyocarditis, vaccine associated complications, vaccine associated myocarditis, covid 19, covid 19 vaccine complication

## Abstract

Perimyocarditis is the inflammation of the pericardium along with the myocardium. Presentation is similar to acute pericarditis, but it is associated with myocardial damage, leading to an elevation in serum troponin and a left ventricular dysfunction (manifested as an ejection fraction of less than 55 percent). Perimyocarditis is mostly managed like acute myocarditis. Etiology is generally idiopathic and likely secondary to viral infections. Cases of vaccine-associated myocarditis have been infrequently reported in past, most recently with the COVID-19 mRNA vaccine. We present a rare case of a young healthy adolescent male who developed perimyocarditis after the first booster dose of the COVID-19 mRNA vaccine.

## Introduction

Inflammation of the pericardium along with the myocardium is called perimyocarditis. It presents as acute pericarditis, associated with an elevation in serum troponin and left ventricular dysfunction from myocardial involvement. Viral infections are the most common etiology for acute perimyocarditis [1]. The COVID-19 mRNA vaccine has been recently noticed to cause myocarditis in rare cases, with risk ranging from 0.0006-0.002% [2,3]. Risks are noticed to be highest among men aged 18-25 years, after their second dose of the mRNA vaccine [4]. Most cases of vaccine-associated myocarditis are usually mild and self-limited. The long-term sequelae of COVID-19 vaccine-induced myocarditis and pericarditis remain unclear. We present a case of a young healthy adolescent male who developed perimyocardits after the first booster dose of the COVID-19 mRNA vaccine.

## Case presentation

A 19-year-old male with no known past medical history presented to the emergency room for evaluation of chest discomfort for two days. He started experiencing burning chest discomfort and pressure at the center of his chest while laying down after eating a fried meal, associated with nausea, which improved with Pepto-Bismol and Tylenol. The discomfort worsened overnight and started radiating down to his bilateral forearms, which woke him up from sleep. He did not report any fever, chills, cough, dyspnea, orthopnea, pedal edema, or recent trauma. He denied any history of smoking, alcohol, or recreational drug use. He received his COVID vaccine booster two weeks ago after which he experienced fevers and fatigue for 1-2 days. The patient reported a history of anaphylaxis to ibuprofen. No hypersensitivity was reported to any past doses of the COVID-19 vaccine (Pfizer-BioNTech) or any other vaccines. Family history was significant for Ehlers-Danlos syndrome in the mother. The patient had never been evaluated for connective tissue disorders.

Vitals were significant for tachycardia with a heart rate of 120 beats per minute. Physical exam significant for an anxious appearance, mild tenderness over the sternum, and hypermobility of joints (particularly fingers). There was no difference in blood pressure in the bilateral upper extremities. High-sensitivity troponin T was elevated at 700 ng/L, followed by 635 ng/L. Erythrocyte sedimentation rate (ESR) was 2mm/hr and C- reactive protein (CRP) was 1.6 mg/L. Urine toxicology was negative. Electrocardiogram (EKG) showed sinus tachycardia, right axis deviation, incomplete right bundle branch block, with some premature ventricular contractions (PVCs). An old EKG from two years ago only showed sinus tachycardia. Telemetry showed rare PVCs and sinus tachycardia. Chest X-ray showed no acute pathology and a normal-sized mediastinum. Bedside ultrasound showed a normal side abdominal aorta. The patient was started on a heparin drip for possible acute coronary syndrome (ACS). Aspirin was not started due to the history of allergy to ibuprofen. Transthoracic echocardiogram showed an ejection fraction (EF) of 60% with no wall motion abnormalities, or valvular abnormalities, and no pericardial effusion was noticed. Coronary angiography was recommended but declined by the patient. Coronary CT angiography (CCTA) was obtained but was limited by motion artifact because of tachycardia. Short segment of the right coronary artery, the distal circumflex, and the branch vessels such as the posterior descending artery could not be satisfactorily evaluated on CCTA but for the most part, the epicardial coronary arteries were widely patent without definite sites of stenosis. 

**Figure 1:**
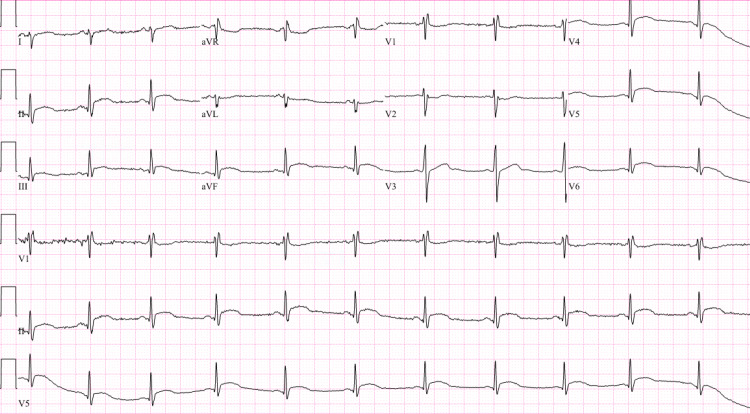
Electrocardiogram showing diffuse ST elevations An electrocardiogram showing sinus tachycardia, right axis deviation, and incomplete right bundle branch block. Diffuse ST segment elevations can be seen in leads II, III, aVF, V4, V5, and V6, which are consistent with pericarditis.

Chest discomfort resolved and the patient was discharged home with oral metoprolol tartrate 25 mg twice daily, with plans for outpatient cardiac MRI. However, he returned to the emergency room three days later with burning abdominal and chest discomfort which was disturbing his sleep. High-sensitivity troponin T this time was elevated at 2862 ng/L. EKG showed ST segment elevations in leads V4, V5, V6, II, III, and aVF (Figure 1). 

**Figure 2:**
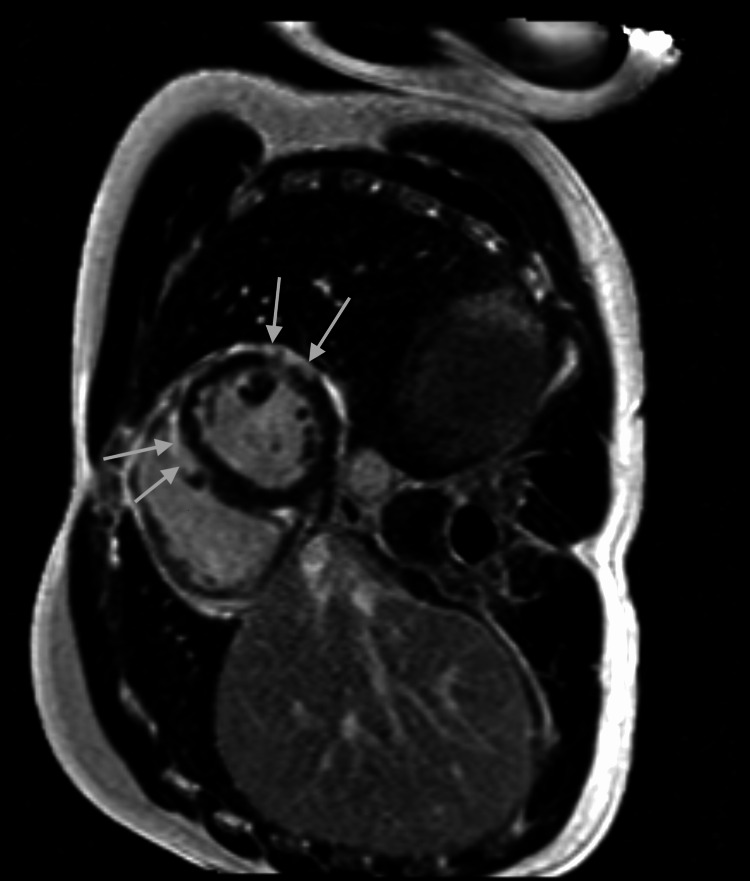
Cardiac MRI showing diffuse subepicardial enhancement of the left ventricular walls with relative sparing of the base, consistent with myocarditis Short-axis cardiac MRI performed at 1.5 T shows myocarditis. The delayed contrast-enhanced image shows diffuse subepicardial enhancement of the left ventricular walls (arrows) with relative sparing of the base.

**Figure 3:**
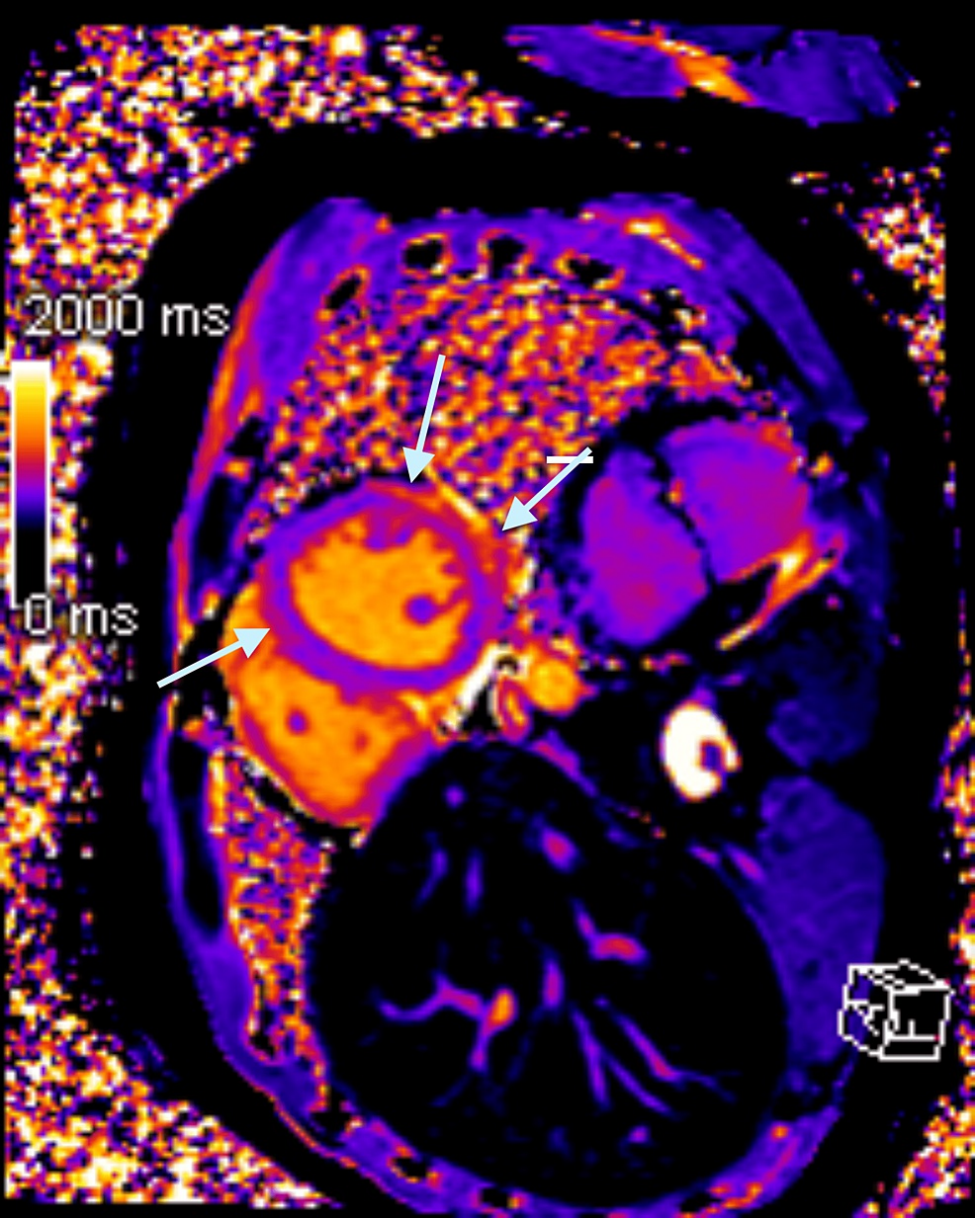
Cardiac MRI demonstrates the prolongation of T1 relaxation time in the subepicardial anterolateral and posterolateral walls, consistent with myocardial edema. Short-axis cardiac MRI performed at 1.5 T shows myocarditis. The native T1 map demonstrates prolongation of the T1 (1250 -1350 msec) relaxation time in the subepicardial anterolateral and posterolateral walls (arrows), consistent with myocardial edema.

**Figure 4:**
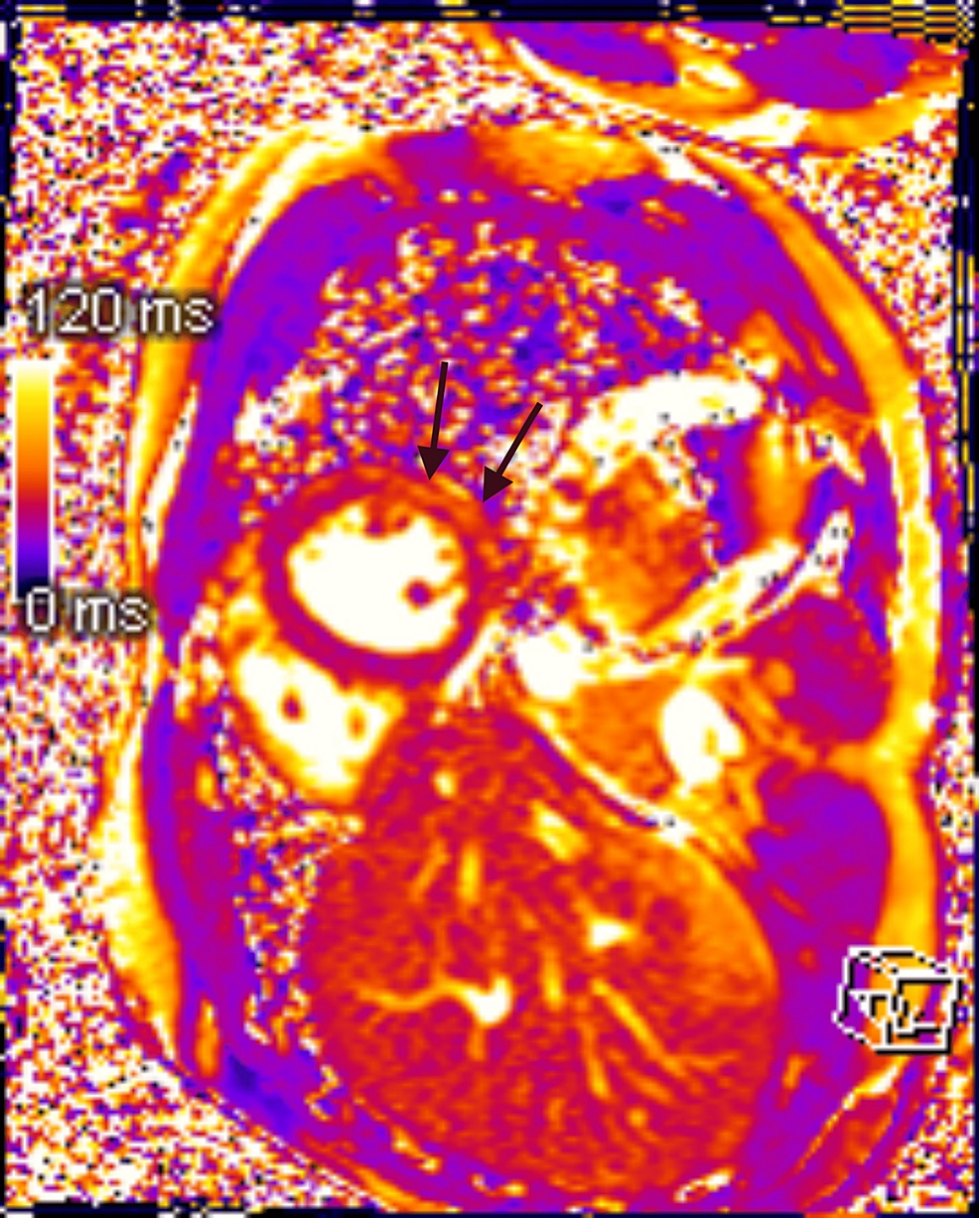
Cardiac MRI demonstrates the prolongation of T2 relaxation time in the subepicardial anterolateral and posterolateral walls, consistent with myocardial edema Short-axis cardiac MRI performed at 1.5 T shows myocarditis. The native T2 map demonstrates prolongation of the T2 (60- 65 msec) relaxation time in the subepicardial anterolateral and posterolateral walls (arrows), consistent with myocardial edema.

A bedside echo showed preserved EF and no wall motion abnormalities. CT angiography of the thorax ruled out an aortic aneurysm or dissection. Infectious panel was negative for acute viral infections, including COVID-19, Epstein-Barr virus, cytomegalovirus, parvovirus, influenza virus, coxsackievirus, human herpesvirus-6, HIV, adenovirus, hepatitis A, hepatitis B, and hepatitis C. Lyme’s antibody panel was negative. Thyroid stimulating hormone was within normal range. The patient was started on colchicine 0.6 mg twice daily, which improved the chest discomfort. Cardiac MRI showed evidence of edema in postero-lateral wall of mild left ventricular (LV) dysfunction with an EF of 44%, a diffuse delayed enhancement of the LV myocardium in a subepicardial distribution, with sparing of the basal segments, consistent with myocarditis (Figure 2-4).

Tachycardia resolved and the patient was noticed to have sinus bradycardia, likely secondary to metoprolol and therefore it was discontinued. He was started on lisinopril 2.5 mg daily. He was discharged home with advice to avoid excessive exertion. The patient continues to follow up with our cardiology clinic, where a follow-up echo one-month post-discharge showed a normal EF. Colchicine was tapered to 0.6 mg daily for a month prior to discontinuation. Lisinopril was also eventually discontinued. The patient continues to be free of any chest discomfort since and can participate in strenuous physical activities without any limitations.

## Discussion

Inflammation of myocardium is referred to as myocarditis. It can involve the myocardium focally or diffusely. Etiology can vary from infectious (viral infections being most common) and noninfectious (for example, hypersensitivity, cardiotoxins, and systemic diseases) [1]. The risk of vaccine-associated myocarditis can range from 0.0006-0.002% with COVID-19 mRNA vaccines [2,3]. Observational studies found a slightly higher risk of myocarditis with the Moderna vaccine, mRNA-1273, when compared to the Pfizer-BioNTech vaccine, BNT162b24. Men aged 18-25 years are at the highest risk after their second dose of mRNA vaccine and this adverse event primarily occurs within 1-7 days of vaccination [4]. Clinical features of myocarditis can be non-specific, such as myalgias, fever, and muscle tenderness. Associated pericarditis can cause chest pain. Fulminant cases can present with a new-onset severe heart failure. Serum troponins are generally elevated. Some patients may also suffer from new arrythmias. Sinus tachycardia is more common than premature atrial complexes, brady-arrhythmias, and atrioventricular blocks. Cases of sudden cardiac deaths secondary to myocarditis have also been reported [5]. The cases of vaccine-associated myocarditis (VAM) are usually mild and self-limited. Cardiac magnetic resonance (CMR) can help diagnose myocarditis but cannot distinguish between the causes of myocarditis. Endomyocardial biopsy can be considered in some select cases with late atrioventricular (AV) block, arrythmias, or unexplained fulminant heart failure [6].

Acute pericarditis is mostly idiopathic and most commonly viral in etiology. Clinical features include pleuritic chest pain, persistent fever, pericardial friction rub, and pericardial effusion [7]. Diffuse ST elevation and PR depression can be seen on EKG. Pericarditis usually has a benign course and low yield of much diagnostic testing. Patients with high-risk features (fever >38^o^C, subacute course, large effusion >20mm on echocardiogram or suspected cardiac tamponade, failure to respond to one week of anti-inflammatory medications, immunosuppression, acute trauma, elevated troponins, or treatment with oral anticoagulants) should be admitted for evaluation and initial treatment [7,8].

Pericarditis and myocarditis can sometimes present together, called perimyocarditis or myopericarditis. Both diagnoses can be associated with a troponin elevation. Myopericarditis is associated with normal LV systolic function, while perimyocarditis leads to a reduced left ventricular ejection fraction (LVEF) of less than 55 percent. Life-threatening causes of elevated troponin (for example ACS, acute aortic syndrome, and pulmonary embolism) should be excluded. Coronary angiography is used in select patients to rule out ACS. Acute phase reactants like ESR and CRP are usually elevated. CMR with late gadolinium enhancement is usually obtained within two weeks of presentation. If CMR is not available, echocardiogram can be utilized. Experts recommend using the updated Lake Louise criteria for CMR diagnosis of myocardial inflammation [9]. Myocardial edema is suggested by the presence of global or regional increase in myocardial T2 relaxation time or an increase in myocardial signal intensity in T2-weighted images. Global or regional increase in myocardial T1, extracellular volume, or late gadolinium enhancement (LGE) is suggestive of myocardial inflammation [9]. Pericardial edema, thickening, LGE, and pericardial effusion on CMR can identify acute pericarditis in cases of myopericarditis [10].

Etiology for perimyocarditis can be idiopathic, infectious (viral being most common), neoplastic, autoimmune, postsurgical, post-myocardial infarction, and vaccine-associated. Vaccine-associated myopericarditis is mostly reported with the smallpox vaccine [11]. Isolated cases have also been reported with Tdap, varicella, influenza, and most recently with the COVID-19 vaccine. However, considering the protective effects of the COVID-19 vaccine against hospitalization and death, and the low incidence of vaccine-associated myocarditis, the overall benefits of the COVID-19 vaccine would still outweigh the risks [12]. Our patient presented with typical pleuritic chest pain two weeks after receiving his first booster dose of the mRNA COVID-19 vaccine (Pfizer-BioNTech vaccine, BNT162b2). EKG showed ST elevation in leads II, III, aVF, V4, V5, and V6, which was consistent with acute pericarditis. Troponin elevation was suggestive of myocardial damage. The patient did not want a cardiac catheterization, but coronary artery disease was ruled out via CCTA. CMR findings of myocardial edema, myocardial inflammation, and LV dysfunction with a reduced EF of 44%, were consistent with acute myocarditis. The patient had an extensive workup, which ruled out any possible infectious causes of perimyocarditis. Endomyocardial biopsy was not considered necessary considering the patent was hemodynamically stable and symptoms improved with colchicine. He could not be started on any Non-steroidal anti-inflammatory drugs (NSAIDs) for acute pericarditis due to prior history of anaphylaxis. Our patient also could not tolerate beta blockers due to bradycardia but was able to continue angiotensin-converting enzyme inhibitors. Fortunately, our patient had a good clinical outcome.

## Conclusions

This case highlights perimyocarditis as a rare complication of mRNA COVID-19 vaccine booster. The long-term sequelae of this vaccine-induced myocarditis and pericarditis remain unclear and further studies are needed. Considering the protective effects of the COVID-19 vaccine against hospitalization and death, and the low incidence of vaccine-associated myocarditis, the overall benefits of the COVID-19 vaccine would outweigh the risks. However, it is unclear if the same rate of benefit is provided by the booster doses of the COVID-19 vaccine. Thus, the decision for booster doses should be individualized based on the risks versus benefits.
